# Optimization of a hatchery residue fermentation process for potential recovery by black soldier fly larvae

**DOI:** 10.1016/j.psj.2025.104946

**Published:** 2025-02-25

**Authors:** Mariève Dallaire-Lamontagne, Yolaine Lebeuf, Linda Saucier, Grant W. Vandenberg, Jérémy Lavoie, Jean-Michel Allard Prus, Marie-Hélène Deschamps

**Affiliations:** a Department of Animal Sciences, Faculty of Agricultural and Food Sciences, Université Laval, 2425 rue de l'Agriculture, Québec, QC G1V 0A6, Canada; b Couvoir Scott, 1798 Route du Président-Kennedy, Scott, QC G0S 3G0, Canada; c Chair of Educational Leadership (CLE) in Primary Production and Processing of Edible Insects, Canada; d Institute of Nutrition and Functional Foods, Faculty of Agriculture and Food Sciences, Université Laval, Quebec, QC G1V 0A6, Canada; e Swine and Poultry Infectious Diseases Research Center, Faculty of Veterinary Medicine, Université de Montreal, Saint-Hyacinthe, QC J2S 2M2, Canada

**Keywords:** Hatchery residues, Fermentation, Microbiological quality, Volatile organic compounds, Black soldier fly

## Abstract

The conventional management of hatchery residues (HR) poses environmental issues and health risks for handlers. This study evaluates the potential of fermentation to reduce pathogens and odors in HR, enabling them to be recovered into feed using black soldier fly. This saprophagous edible insect is valued for its ability to efficiently bioconvert organic residues into high-quality biomass. Due to the low carbohydrate content of HR, whey permeate was added at lactose inclusion levels of 0, 5, 15, 25, and 35% (dry basis) to optimize fermentation. Using a commercial ferment starter culture (0.3%, wet basis), HR were fermented under semi-anaerobic conditions for two weeks. Fermentation metrics, including pH, microbiological loads (total aerobic mesophilic, presumptive lactic acid bacteria, coliforms, *Escherichia coli*), volatile fatty acids, and volatile organic compounds, were monitored at days 0, 3, 7, and 14. Optimal stabilization was achieved with lactose inclusion of 15 to 35% after 7 days, which reduced pH (<5.3), increased lactic (87.82 mg/g) and acetic (20.28 mg/g) acid production, and decreased coliform and *Escherichia coli* counts below detection limit (1.7 log cfu/g). The production of compounds associated with unpleasant odors was also limited. The use of a ferment did not result in a greater reduction of coliform counts, the initial loads of lactic acid bacteria (> 7 log cfu/g) being sufficient to initiate spontaneous fermentation. However, ferment was found to be efficient in heated HR. These findings demonstrate the effectiveness of fermentation for stabilizing HR, highlighting its potential for integration into insect bioconversion systems.

## Introduction

The management of livestock residues using conventional methods such as thermal rendering or composting is associated with environmental, social, and economic issues. Residues may contain pathogenic microorganisms (Hutchison et al., 2005) and be associated with unpleasant odors that can adversely affect the health and well-being of human communities surrounding production and management sites (Sazakli and Leotsinidis, 2021). Although thermal rendering enables livestock residues to be transformed into feed ingredients, this option can be expensive for producers and involves significant energy costs and greenhouse gas emissions (Gooding, 2012). In the case of composting, carcasses and manure are recycled into organic fertilizer by microbial activity. However, composting does not ensure optimal reuse of the nutrient content of these residues in accordance with the 3Rs (reduce, reuse, recycle) principle (Gouvernement du Québec, 2022).

Among livestock wastes, hatchery residues (HR), such as unmarketable chick carcasses, discarded eggs and eggshells (Orrico et al., 2020), are particularly complex to manage in view of their high microbiological loads (5.4 to 8.5 log cfu/g), including pathogens such as *Salmonella* spp. or *E. coli,* and the resulting putrid odors (Dallaire-Lamontagne et al., 2024). In Quebec, HR are usually recovered at high cost (244$/t in 2022; Couvoir Scott Ltee, Scott, Qc, Canada, personal communication) by rendering companies. Yet, HR is an energetic co-product with high protein (21.3 to 49.4%) and lipid (14.6 to 29.1%) contents (Dallaire-Lamontagne et al., 2024) and would benefit from being valorized more efficiently. Depending on regulatory restrictions in different parts of the world (Lähteenmäki-Uutela et al., 2018), HR could be used as a rearing substrate for black soldier fly larvae (BSFL; *Hermetia illucens* (L.), Diptera: Stratiomyidae) (Zhang et al., 2021; Dallaire-Lamontagne et al., 2024). This species of saprophagous edible insect can be reared at large scale to produce pet food (Bosch and Swanson, 2020) and feed for livestock (Veldkamp and Vernooij, 2021). Even though the use of processed animal products as a substrate for rearing edible insects is prohibited in the European Union (Regulation (EC) No 1069/2009 IPIFF, 2024), there are currently no such limitations in Canada (Larouche et al., 2023). Properly processed, this could favour the application of entomotechnologies for the recovery of livestock residues (Dallaire-Lamontagne et al., 2024). However, it will depend on the ability to demonstrate to government agencies that the process and product safety can be ensured, with mitigating risks upstream being essential to prevent further contamination in the production chain.

Fermentation pre-treatment could be a passive and promising option to stabilize HR (Deshmukh and Patterson, 1997a, b; Baba et al., 2022) without significantly affecting their proximate composition (Cai et al., 1994). Lactic acid fermentation has long been used to prepare a wide range of traditional food products such as cheese, yogurt (Solomons, 2002), fermented sausage (Wang et al., 2013) as well as feed (Raa and Gildberg, 1982; Eissa et al., 2022). Pathogenic risks can be reduced through the acidification and activity of lactic acid bacteria (LAB) (Reis et al., 2012; Bartkiene et al., 2019). Indeed, LAB produces organic acids and antimicrobial compounds (e.g., bacteriocins) which contribute to the acidification of the fermented substrates and inhibition of pathogenic microorganisms (Castellano et al., 2008; Castilho et al., 2019) associated with the release of putrid odors (Casaburi et al., 2015).

The yield of the fermentation process depends on the presence of fermentable sugars, which are found in very small quantities, if at all, in hatchery residues (0-15.3%; Dallaire-Lamontagne et al., 2024). Deshmukh and Patterson (1997a, b) have showed that HR could be efficiently fermented in anaerobic conditions over 21 days, using a constant ratio chicks and shell waste ratio (60:40), a commercial ferment and chocolate as a source of carbohydrates. The technical feasibility of the implementation of such fermentation depends on several factors such as temporal fluctuations in nutritional and microbiological contents of HR, number and size of fermentation units; use of ferment; access to low-cost and local carbohydrates sources (Bruins and Sanders, 2012; Samer and Abdelsalam, 2019; García-Díez and Saraiva, 2021).

The aim of this project was thus to optimize a HR fermentation process to efficiently control the microbiological and odor risks upstream of BSFL bioconversion. The hypothesis was that the addition of ferment, along with optimal carbohydrate inclusion rates and fermentation duration, would improve the microbiological quality of HR and reduce the production of volatile organic compounds associated with HR odors.

## Materials and methods

The trials were conducted three times (*n* = 3) over a period of six months to ensure the reliability and reproducibility of the results, despite temporal fluctuations in HR composition. Each trial adhered the same experimental protocol, which included the following procedures:

### Hatchery residues

The hatchery residues (cracked, infertile, infected, or unhatched eggs, as well as eggshells and unmarketable chick carcasses) were provided by an industrial partner (Couvoir Scott, Scott, Qc, Canada). Residues were collected into a 12-ton capacity silo outside of the hatchery and stored for a maximum of 24 h. For each trial, 10 kg of HR were sampled in sealed plastic containers (24.6 L, S-15637BLU, Uline, Pleasant Prairie, WI), ground and homogenized using a meat grinder (#12 Big Bite Meat Grinder, LEM, ¾ HP, OH), before being stored overnight at 4°C. Dry matter (DM) content of HR was estimated after drying a sub-sample of 2 g overnight at 100°C (VWR® Forced Air Ovens, VWR International, Radnor, PA) according to an adapted method combining the ones proposed by Ahn et al. (2014) and Shreve et al.(2006).

### Experimental design

A freeze-dried commercial ferment designed for the fermentation of meat products (LALCULT® Protect SAX-01, Lallemand, Montréal, Canada), containing *Pediococcus acidilactici, Staphylococcus xylosus, Lactobacillus sakei*, and *Debaryomyces hansenii,* was used as a starter culture at an inclusion level of 0.3% (1.62 × 10^8^ cfu/g, wet basis). The ferment was pre-activated for 1 h in distilled water at a 1:10 dilution before inoculation into the HR. For each trial, 190 g of HR (dry basis) was placed in commercial fermentation jars (*n* = 27) with removable inner lid to create semi-anaerobic conditions (3.4 L, Premium E-jen Kimchi Fermentation & Storage Container, Crazy Korean Cooking, Ridgefield Park, NJ). Lactose was inoculated into the HR mixtures at inclusion levels (IL) of 0, 5, 15, 25 and 35% (dry basis of HR) using a dry (D; 96.2% DM) or wet (W; 45% DM) source of whey permeate, two main co-products of the Quebec dairy industry (Agropur, Saint-Hyacinthe, Qc, Canada).

Nine substrates (Su) based on raw (S) or inoculated (0.3%, wet basis) hatchery residues, without heat pre-treatment (F) or with heat pre-treatment (T), and supplemented with different levels of lactose inclusions (0, 5, 10, 25, 35%, dry basis) from a dry (D) or wet (W) source of whey permeate where compared:

• S (spontaneous fermentation) consisted of raw HR without addition of a ferment starter culture.
• F (inoculated fermentation) consisted of raw HR with ferment (0.3%, wet basis).
• T (thermal pre-treatment) consisted of thermally treated HR with ferment (0.3%, wet basis).

The T substrates were prepared by placing the HR in vacuum-sealed bags (Smooth Vacuum Bags SB Series, 12″ x 16″, Sous Vide Premium, Montréal, Canada) using a vacuum sealer (Eco Vacuum, Orved Spa, Venice, Italy). These bags were then placed in a pressure cooker (21.8 L; Induction Compatible Pressure Canner 01784, National Presto Industries, Eau Claire, WI) at a high pressure (15 psi; 121°C) for 10 min to reduce LAB loads below 2 log cfu/g. Once the HR was cooled down and reached room temperature, the ferment was added according to the same inoculation parameters as the F substrate.

Fermentation jars were closed by adjusting the under-lid to the level of the substrate to limit the contact with air. A small opening (0.8 cm diameter) in the sub-lid allowed gases accumulation during the fermentation process. The jars were then transferred to Université Laval (Quebec, Qc, Canada) and stored for 14 days at 21°C.

### Sampling and monitoring

Fermentation was monitored at day (d) 0, 3, 7 and 14, under a chemical hood to control the odors using the same jars throughout the experiments. To limit disruption of fermentation due to prolonged exposure to oxygen, fermentation jars were opened for a maximum of 5 min to manually stir the substrates, take measurements (pH, temperature, chroma color, and glucose content) and to collect samples for subsequent analyses. Moreover, the jars were closed at the end of each of the four measurement and sampling periods by adjusting the inner lid to the level of the substrate and evacuating the air.

Substrate pH and temperature were measured using a portable pH meter (Orion Star™ A221, Thermo Fisher Scientific, Waltham, MA) equipped with a probe (Orion™ Triode™ 9107BNMD, Thermo Fisher Scientific, Waltham, MA). Residue color was also assessed to evaluate changes in substrate conformation over time using a colorimeter (chromameter CR400/410, Konica Minolta, Tokyo, Japan) equipped with a conical open port, an 8 mm aperture, a diffuse illumination, a 0° viewing angle geometry and a D65 light source according to the reflectance coordinates (L*,a*,b*; CIE 1976). The color intensity (chroma, C*; equation (1)) was used to compare the samples (International Commission on Illumination, 2004).

(1) $$C^{*}=\;\sqrt{a^{*2}+b^{*2}}$$

The glucose content of HR was estimated using colorimetric strips (MilliporeSigma™ MQuant™ Glucose Test Strips, Burlington, MA).

Samples were taken for microbiological analyses (25 g), volatile fatty acid (VFAs, 1 g) content and volatile organic compound (VOCs, 10 g). Microbiological samples were stored at −80°C to minimise microbial cell inactivation (not assessed) resulting from the formation of ice crystals. VFAs and VOCs samples were stored at −20°C.

### Microbiological analyses

For each sample taken at every day of monitoring over the three trials (*n* = 324), microbial analyses were conducted in duplicate for total aerobic mesophilic (TAM), presumptive lactic acid bacteria (LAB) and coliforms (COL), as an indication of overall contamination of HR during the fermentation process. Frozen samples (25 g) were thawed at 4°C for 12 h and were homogenized in 225 ml of buffered peptone water (Difco Laboratories Inc., Becton Dickinson, Franklin Lakes, NJ) using a Stomacher (Stomacher® 400C, Seward Laboratory Systems Inc., London, UK) for 120 s at 230 RPM. Serial dilutions (1:10) were made in peptone water (0.1%) for enumeration on agar plates (Saucier et al., 2000). Counts of TAM were performed on Plate Count Agar medium (Difco Laboratories Inc.) after being incubated at 35°C for 48 h (MFHPB-18; Health Canada, 2001). Presumptive LAB were enumerated on de Man, Rogosa, and Sharp agar (Difco Laboratories Inc.) after an incubation at 25°C for 48 h under anaerobic conditions (GasPak EZ container system, Becton Dickinson, Franklin Lakes, NJ; Saucier et al., 2000). The COL and *E. coli* counts were enumerated from selective petrifilms (3M™ Petrifilm™ Rapid *E. coli*/Coliform Count Plate, 3M, Montréal, Canada) after being incubated at 35°C for 24 h (MFHPB-34; Health Canada, 2016).

Colony-forming (cfu) units were log-transformed per gram of substrate (log10 cfu/g, wet basis) before statistical analyses (Gill, 2000). The detection threshold was defined based on the value corresponding to the presence of a single colony on one of the two duplicates plates. It was used for statistical analysis when no colonies were detected on both plates for a given sample (Saucier et al., 2021).

### Volatile compounds analyses

#### Volatile fatty acids (VFAs)

To measure the concentration of volatile fatty acids (VFAs) in the substrates (lactic, acetic, propionic, butyric and isobutyric acids), samples were thawed at 4ºC overnight and solubilized in a solution of H_2_SO_4_ (1.5%) at a 1:3 dilution. Acidified samples were centrifuged at 10 000 RPM at 4°C for 15 min followed by centrifugation of supernatants at 15 000 RPM at 4°C for 10 min. The supernatants were mixed with 0.2 g of resin (AG 50WX8, 100–200 Mesh, H, BioRad, Hercules, CA) and filtered (polyethersulfone filter; pore size = 0.2 μm; diameter = 25 mm). The VFAs were then determined by high pressure liquid chromatography using analytical HPLC Systems (1260 Infinity II LC System; Agilent, Santa Clara, CA) as described by Benchaar et al. (2013).

#### Volatile organic compounds (VOCs)

Various VOCs associated with unpleasant odors (dimethyl sulfide, dimethyl disulfide, dimethyl trisulfide, trimethylamine, indole) were measured in non-heat-treated HR, for treatments from 0 to 25% IL (W), at 0 and 14 days of fermentation. Analysis of the VOC concentrations in the HR was carried out using the solid phase microextraction (SPME) technique with a Combi PAL autosampler (CTC Analytics, Zwingen, Switzerland) connected to the GC Agilent 8890 GC and the MS Agilent 5977B GC/MSD (Agilent Technologies Canada Inc., Mississauga, Canada). For the analyses, 0.5 g of HR sample and 500 µl of an internal standard of hexanal-δ12 (1.5 µg/ml) were placed in a 20 ml glass vial and sealed with a cap having a white PTFE/blue silicone septum. The VOCs were extracted into the headspace with Supelco 57299-U SPME fibre (50/30 µm DVB/CAR/PDMS, 2 cm StableFlex, 23 Ga Gray-Notched Autosampler; Supelco Inc., Bellefonte, PA). The samples were pre-equilibrated for 5 min at 40°C with 250 RPM agitation. The fibre was then exposed in the headspace at the same temperature for 5 min. Once the extraction was complete, the VOCs were extracted with the SPME fibre were thermally desorbed in the GC injector in splitless mode at 255°C for 90 s. Between each sample, the fibre was conditioned at 270°C for 30 min. The VOCs were separated using the Optima Wax column (60 m x 250 µm x 0.25 µm; Macherey-Nagel, Düren, Germany). The GC oven temperature was maintained at 37°C for 2 min and then increased at a rate of 5°C/min to a temperature of 250°C. At the end, the oven temperature was maintained at 250°C for 15 min. The helium flow rate was 0.7 ml/min. The mass spectrometer (MS) operated in electron impact ionization mode at 70 eV, with a mass-to-charge ratio (m/z) range of 35 to 350. The data acquisition mode was scanned. The temperatures of the MS transfer line and the source were 255°C and 230°C, respectively. The VOCs were then identified by comparing their mass spectra using authentic standards from Sigma Aldrich (Oakville, Canada) and/or using the National Instituted and Technology (NIST) mass spectral database (NIST MS search version 2.3, Gaithersburg, MD). The relative concentration of each compound was determined by dividing the peak area of the compounds of interest by the peak area of the hexanal-δ12 internal standard and multiplying this ratio by the concentration of the internal standard (expressed in µg/kg). The hexanal-δ12 standard was obtained from C/D/N Isotopes Inc (Pointe-Claire, Canada).

### Statistical analyses

Statistical analyses were performed using R studio software (version 4.2.2). Data from repetitions of the experiment over three different trials were analysed using a 3-factor mixed linear model (Substrate (Su), Lactose inclusion level (IL) and days of fermentation (d)). A repeated measures ANOVA over time with Tukey correction was applied to compare the means of the different groups. When significant differences at a confidence level of 0.95 (alpha = 0.05) were obtained, a multiple comparison test (Tukey's range test) was applied. The normality of the variance was verified using the Shapiro-Wilk test and the homogeneity of the variance was visually confirmed by a graph of the scaled residuals and predicted values. Figures, including a principal component analysis model (PCA) to visualize interactions among the parameters measured during the fermentation of HR over three trials (*n* = 81) and Pearson correlation coefficients (r) were generated using JMP statistical software (JMP Student Subscription 17.2.0).

## Results

Fermentation of HR modified their physico-chemical properties (pH, colour, VFAs and VOCs) and their microbiological loads (TAM, LAB, COL and *E. coli*) according to the fermentation conditions. In particular, the addition of a ferment, the application of a preheat treatment and the IL led to variations in the characteristics of the fermented HR. However, for all the parameters, the form in which the whey permeate was used (D or W) had no significant influence on the variations measured.

### pH and glucose content

**Fig. 1:**
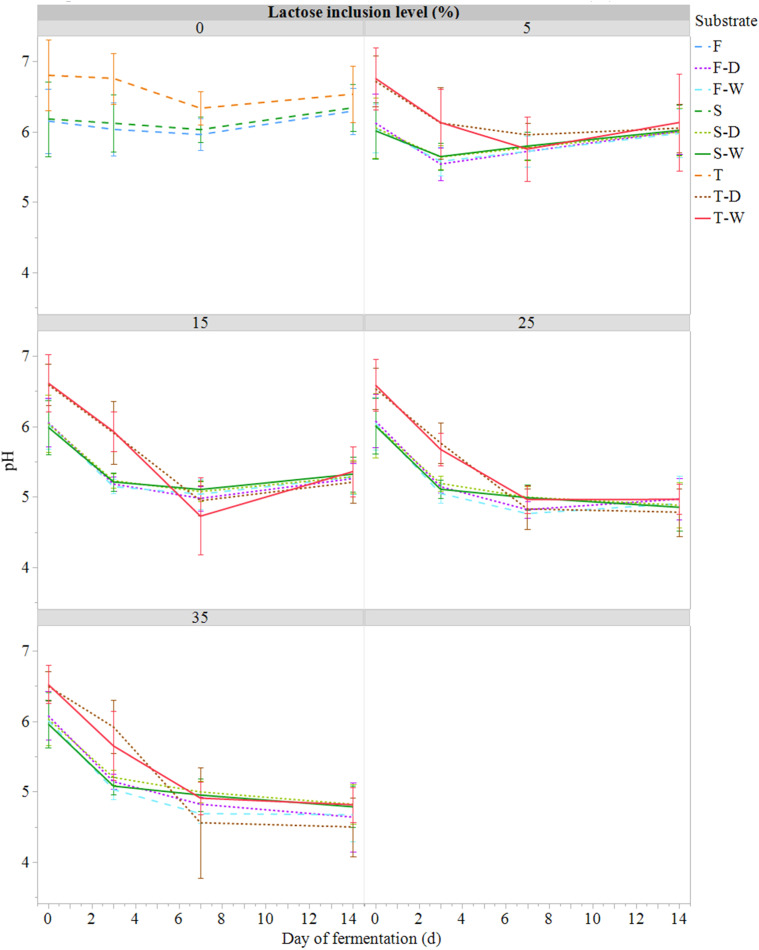
Evolution of pH (mean ± SE; *n* = 81) during semi-anaerobic fermentation (14 days at 21°C) of raw (S) or ferment-inoculated (0.3%, wet basis) hatchery residues, without heat pre-treatment (F) or with heat pre-treatment (T), with different lactose inclusion level (0, 5, 15, 25, 35%, dry basis) using a dry (D) or wet (W) source of whey permeate.

The evolution of HR pH over the fermentation period is presented in Fig. 1. At days 0 and 3, the pH of T substrates was significantly higher (*P* < 0.001) than for F and S substrates, but after 7 days there was no significant difference between all the substrates for the same IL. An effect of the IL was measured for 15 to 35 % treatment, for which the pH was significantly lower (*P* < 0.001) than for the 0 and 5 % IL, from days 3 and 7, respectively. At day 7, the pH of 15 to 35% IL decreased to value around five. However, at day 14, the pH of the 15% IL treatment increases (5.29 ± 0.23) compared to the 25-35% IL, which remained under 5.10 (4.80 ± 0.30).

The estimated glucose content of the HR tended to decrease over time until it could no longer be detected after 3, 7 and 14 days for treatments of 0 to 5%, 15% and 25-35% IL, respectively.

### Microbiological loads

**Fig. 2:**
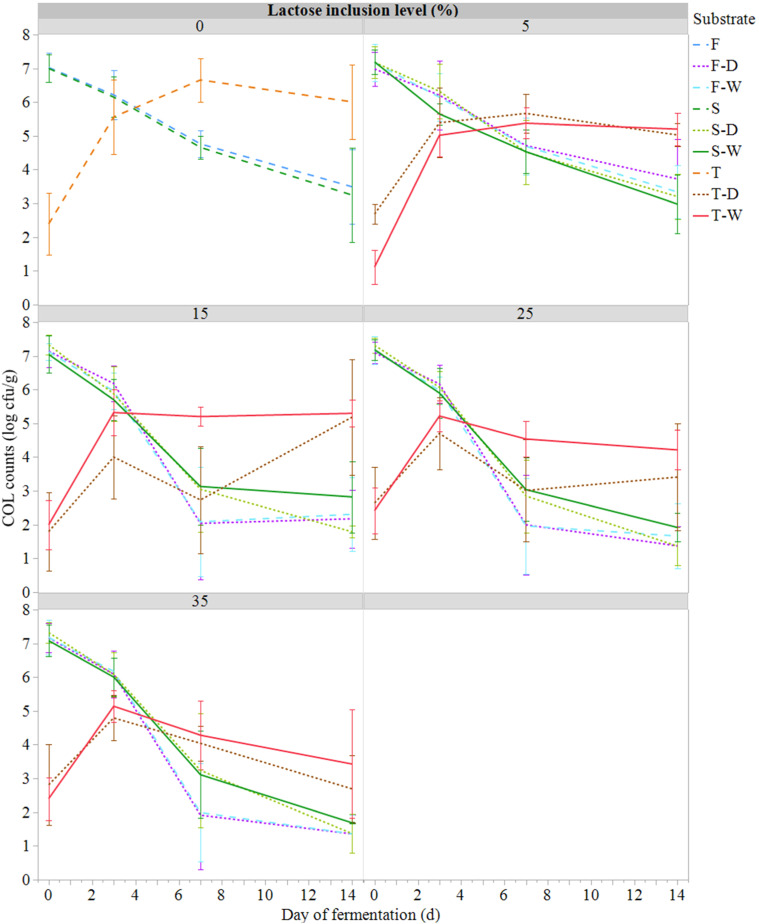
Evolution of the coliform counts (COL, log cfu/g; mean ± SE; *n* = 81) during semi-anaerobic fermentation (14 days at 21°C) of raw (S) or ferment-inoculated (0.3%, wet basis) hatchery residues, without heat pre-treatment (F) or with heat pre-treatment (T), with different lactose inclusion level (0, 5, 15, 25, 35%, dry basis) using a dry (D) or wet (W) source of whey permeate.

**Fig. 3:**
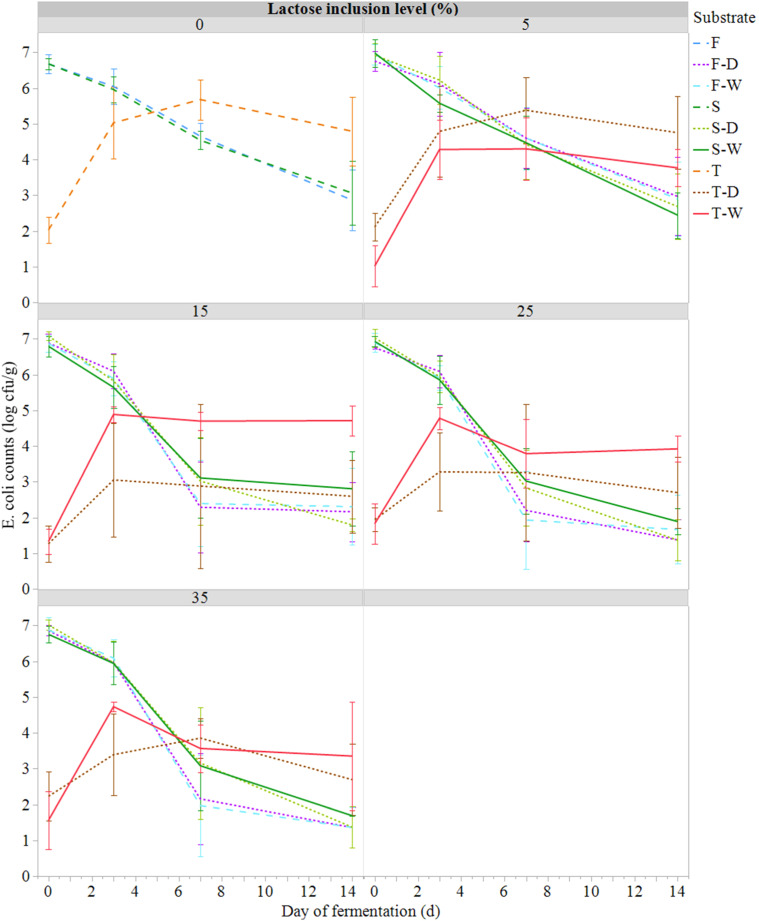
Evolution of *Escherichia coli* counts (*E. coli*, log cfu/g; mean ± SE; *n* = 81) during semi-anaerobic fermentation (14 days at 21°C) of raw (S) or ferment-inoculated (0.3%, wet basis) hatchery residues, without heat pre-treatment (F) or with heat pre-treatment (T), with different lactose inclusion level (0, 5, 15, 25, 35%, dry basis) using a dry (D) or wet (W) source of whey permeate.

The application of different substrates and IL influenced COL and *E. coli* loads (Fig. 2, Fig. 3). From the first day of fermentation, the COL counts (which include *E. coli*) of substrates T were significantly lower (2.3 ± 0.3 log cfu/g) compared with substrates that had not undergone heat treatment (7.1 ± 0.1 log cfu/g). However, while the COL counts of the F and S substrates tended to decrease over the days of fermentation until they reached an average of 2.4 ± 1.0 log cfu/g, those of the T substrates increased to reach an average of 4.7 ± 1.2 log cfu/g at day 14. Substrates F also showed an accelerated reduction in COL counts over time, reaching values below the detection threshold of 1.7 log cfu/g after day 7 for treatment with 25 to 35% IL compared with substrates S who reach average value around 3 log cfu/g for the same IL. This reduction in cell counts was significant both statistically (*P* < 0.001) and biologically (variation of more than 1 log unit). Similarly, after 7 days of fermentation, 15 to 35 % IL resulted in a significantly lower COL counts compared with 0 to 5% IL treatments. Similar results were obtained for *E. coli*.

**Fig. 4:**
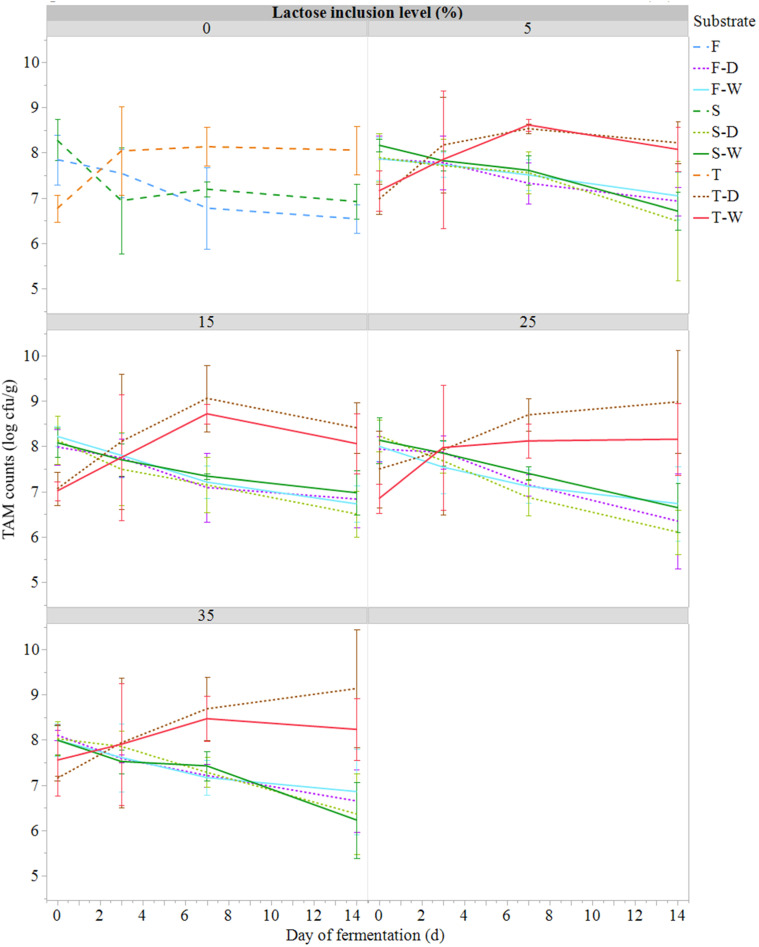
Evolution of total aerobic mesophilic counts (TAM, log cfu/g; mean ± SE; *n* = 81) during semi-anaerobic fermentation (14 days at 21°C) of raw (S) or ferment-inoculated (0.3%, wet basis) hatchery residues, without heat pre-treatment (F) or with heat pre-treatment (T), with different lactose inclusion level (0, 5, 15, 25, 35%, dry basis) using a dry (D) or wet (W) source of whey permeate.

**Fig. 5:**
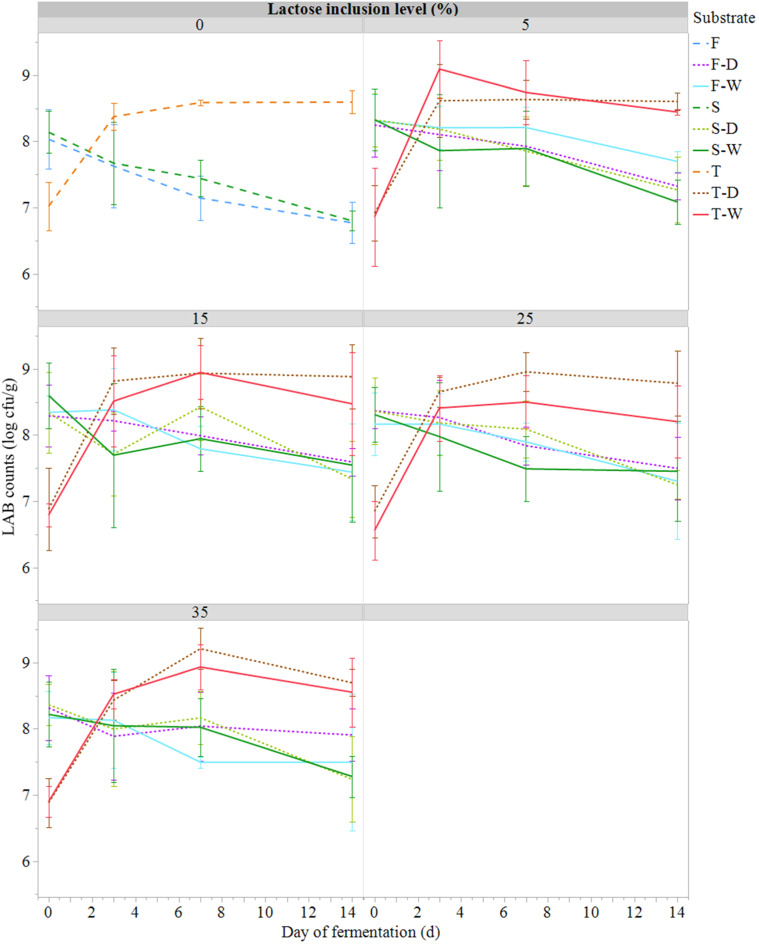
Presumptive lactic acid bacteria counts (LAB, log cfu/g; mean ± SE; *n* = 81) during semi-anaerobic fermentation (14 days at 21°C) of raw (S) or ferment-inoculated (0.3%, wet basis) hatchery residues, without heat pre-treatment (F) or with heat pre-treatment (T), with different lactose inclusion level (0, 5, 15, 25, 35%, dry basis) using a dry (D) or wet (W) source of whey permeate.

The LAB and TAM counts measured (Fig. 4, Fig. 5) were found to be less variable than for COL and *E. coli* over the fermentation period and between treatments (Fig. 2, Fig. 4). The T substrates had lower initial LAB and TAM counts than the F and S substrates, but then higher loads at day 14. Indeed, the initial LAB and TAM counts in F and S substrates were high (8 to 9 log cfu/g; Fig. 3, Fig. 4). The addition of ferment in T substrates led to LAB counts of 7 log cfu/g while the addition of ferment in F substrates did not elevate the initial load of LAB compared to the S substrates.

### Production of volatile fatty acid compounds (VFAs)

**Fig. 6:**
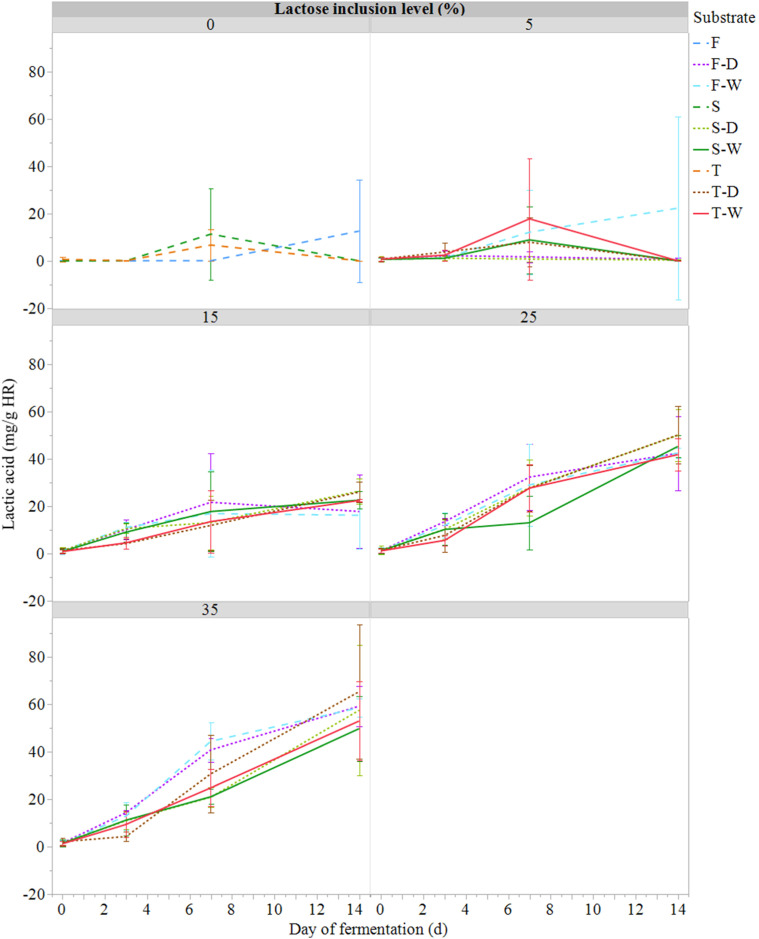
Lactic acid concentration (mg/g; mean ± SE; *n* = 81) during semi-anaerobic fermentation (14 days at 21°C) of raw (S) or ferment-inoculated (0.3%, wet basis) hatchery residues, without heat pre-treatment (F) or with heat pre-treatment (T), with different lactose inclusion level (0, 5, 15, 25, 35%, dry basis) using a dry (D) or wet (W) source of whey permeate.

Lactic acid was the main VFA generated during fermentation. After 3 days, its concentration started to be significantly higher in the 25 to 35% IL compared to the 0 % IL treatments (*P* < 0.001; Fig. 6). It was also observed that a distinct acidic odor was released from these fermentation units (substrates at 25 to 35% IL) when opened at day 14. Other types of VFAs, such as acetic (20.28 mg/g), propionic (14.01 mg/g), butyric (8.54 mg/g), and isobutyric (0.94 mg/g) acids, were produced during HR fermentation, though to a lesser extent than lactic acid (87.82 mg/g), which exhibited the highest maximum concentration (HR, dry basis) among them.

### Interactions between parameters

The different parameters measured were influenced by the fermentation conditions (Su, IL, d). These interactions are presented in Table 1. The effect of Su on pH, Chroma, TAM, LAB, COL, *E. coli* counts, and butyric acid concentration varied between days (*P* < 0.001). The same applies to the IL, whose effects on pH, Chroma, COL, *E. coli* counts, and lactic acid concentration differ according to the days of fermentation (*P* < 0.001).

**Table 1:** Interactions (P-value) between the physico-chemical and microbiological characteristics as a function of substrate (Su), lactose inclusion level (IL) and days of fermentation (d).

	Substrate (Su)	Inclusion level (IL)	Days (d)	Su*IL	Su: d	IL: d	Su:IL: d
pH	<0.001	<0.001	<0.001	0.996	<0.001	<0.001	0.100
Chroma	<0.001	<0.001	<0.001	0.150	<0.001	<0.001	0.701
TAM	<0.001	0.706	<0.001	0.789	<0.001	0.999	0.967
LAB	<0.001	<0.001	<0.001	0.392	<0.001	0.860	0.975
Coliforms	<0.001	<0.001	<0.001	0.150	<0.001	<0.001	0.701
*Escherichia coli*	<0.001	<0.001	<0.001	0.017	<0.001	<0.001	0.928
Lactic	0.545	<0.001	<0.001	0.991	0.851	<0.001	0.763
Acetic	<0.001	0.011	<0.001	0.300	0.169	0.997	0.774
Propionic	<0.001	0.114	<0.001	0.627	0.089	0.881	0.585
Butyric	<0.001	<0.001	<0.001	0.823	0.035	0.567	0.480
Iso-Butyric	<0.001	<0.001	<0.001	0.722	0.060	0.219	0.581

TAM = total aerobic mesophilic; LAB = presumptive lactic acid bacteria; Lactic = lactic acid; Acetic = acetic acid; Propionic = propionic acid; Butyric = butyric acid; Iso-Butyric = Iso-Butyric acid.

**Fig. 7:**
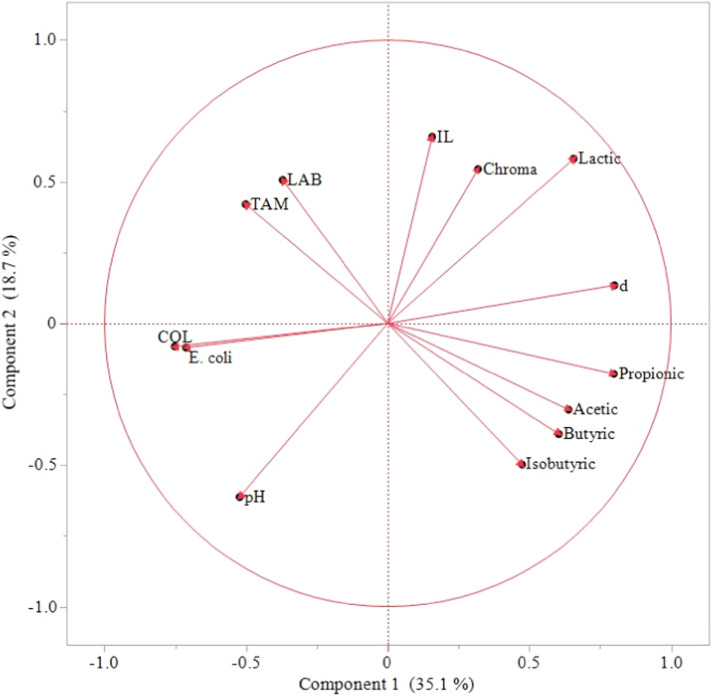
Principal component analysis (PCA) of parameters measured during hatchery residue fermentation over three trials (*n* = 81). The model transposes 53.8% of the variability of the measured parameters with Component 1 and 2. IL = lactose inclusion level (%); *d*= days of fermentation; TAM = total aerobic mesophilic; COL = coliforms; LAB = presumptive lactic acid bacteria.

In addition to the fermentation conditions, some of the measured parameters were highly correlated to each other (Table S1 in Supplementary material). These interactions can be visualized using PCA (Fig. 7). Lactic acid concentration and Chroma were inversely correlated with pH (*r* = 0.36) as well as with COL and *E. coli* counts (*r* = −0.52 and −0.48 respectively), indicating that higher lactic acid production and intensification of HR color is linked to acidification of the substrate and lower loads of these microorganisms. Similarly to COL and *E. coli* counts (*r* = 0.96), TAM and LAB counts were strongly correlated with each other (*r* = 0.71).

### Production of volatile organic compounds (VOCs)

The VOC concentrations obtained were highly variable, both between the different trials carried out and the substrate (Su) (Table 2). Although no significant differences were measured over time for a given treatment, VOC concentrations appear to be more stable at higher IL. Indeed, at 14 days, dimethyl disulfide, dimethyl trisulfide, trimethylamine and indole concentrations were significantly higher for 0% than for 15 to 25% IL treatments, regardless of Su.

**Table 2:** Initial and final volatile organic compounds (VOCs; µg/kg; mean ± SE) after semi-anaerobic fermentation (14 days at 21°C) of raw (S) or inoculated (0.3%) hatchery residues after (T) or without (F) heat treatment and supplemented with different levels of lactose inclusions (IL; 0, 5, 10, 25, 35%, dry basis) using a dry (D) or wet (W) source of whey permeate.

VOCs	IL	Substrates	d	
			0	14
Dimethyl sulfide	0	F	1.37 ± 0.12	1.47 ± 0.12
		S	1.38 ± 0.12	1.42 ± 0.12
	15	F-W	1.35 ± 0.11	1.33 ± 0.11
		S-W	1.42 ± 0.11	1.34 ± 0.11
	25	F-W	1.38 ± 0.13	1.32 ± 0.13
		S-W	1.40 ± 0.13	1.34 ± 0.13
Dimethyl disulfide	0	F	757.9 ± 1161.4	6552.1 ± 4701.3 ^a^
		S	540.5 ± 828.4	8072.4 ± 5792.1 ^a^
	15	F-W	66.3 ± 101.7	809.1 ± 580.6 ^b^
		S-W	183.8 ± 281.8	1313.8 ± 942.8 ^b^
	25	F-W	57.9 ± 88.9	495.8 ± 355.8 ^b^
		S-W	109.5 ± 168.0	942.7 ± 676.4 ^b^
Dimethyl trisulfide	0	F	107.3 ± 143.6	2666.6 ± 1591.9 ^a^
		S	67.2 ± 89.9	1994.1 ± 1190.5 ^a^
	15	F-W	10.0 ± 13.5	32.0 ± 19.1 ^b^
		S-W	34.4 ± 46.1	70.0 ± 41.9 ^b^
	25	F-W	16.6 ± 22.4	43.9 ± 26.2 ^b^
		S-W	22.8 ± 30.6	42.4 ± 25.4 ^b^
Trimethylamine	0	F	32864 ± 24150	172169 ± 135972 ^a^
		S	28823 ± 21205	95678 ± 75656 ^a^
	15	F-W	11206 ± 8363	12581 ± 10131 ^b^
		S-W	15815 ± 11723	7966 ± 6492 ^b^
	25	F-W	6020 ± 4583	4382 ± 3666 ^b^
		S-W	10642 ± 7952	6611 ± 5423 ^b^
Indole	0	F	4838 ± 4063	52948 ± 23040 ^a^
		S	4947 ± 4153	32404 ± 14111 ^a^
	15	F-W	1468 ± 1268	9539 ± 4173 ^b^
		S-W	2525 ± 2144	10288 ± 4498 ^b^
	25	F-W	1700 ± 1460	5718 ± 2512 ^b^
		S-W	1800 ± 1543	6362 ± 2792 ^b^

VOCs; EM mean ± SE, µg/kg. For each type of VOC measured, different letters on a given day of fermentation (d; 0 or 14) indicate significant differences (*p* < 0.05) between treatments (IL and Substrate).

## Discussion

The results obtained showed that lactic acid fermentation under optimized conditions could effectively stabilize HR and reduce the risks of introducing microbiological contaminants into the BSFL production chain. Indeed, when fermentation of raw HR was carried out over a period of 7 to 14 days and initiated by lactose inclusion at 25 to 35% IL, the pH was reduced below a value of 5 and the microbiological risks associated with the presence of COL and *E. coli* were mitigated. Previously, several authors have demonstrated the effectiveness of fermentation to acidify and bring bioprotective effects to meat and egg products. Fermentation of pork meat with *Lactobacillus spp.* demonstrated inhibitory effects against *Clostridium spp.* (Di Gioia et al., 2016), while the fermentation of ostrich meat inhibited *Listeria monocytogenes* through bacteriocins like plantaricin and curvacin produced by the starter cultures (Dicks et al., 2003). However, as Gram-negative bacteria are generally resistant to the action of bacteriocins from LAB (Prudêncio et al., 2015), the variations in COL and *E. coli* counts measured in HR must be attributed to other mechanisms. In addition to the production of bacteriocins, food fermentation also generates organic acids which contribute to the acidification of the substrate. LAB tolerate weak lipophilic acids and produce them during metabolism, inhibiting spoilage microorganisms when sufficient quantities are generated rapidly during fermentation (Reis et al., 2012). For example, fermenting egg albumen at a basic pH significantly reduced microbial loads within 30–36 h due to acidification Nahariah et al. (2015). Similarly, fermentation of mealworm paste with lacto-fermenting bacteria lowered the pH from 6.7 to 5.1 in 3 days Borremans et al. (2020a). In the case of HR fermented with lactose inclusion rates of 15-35%, the high proportion of lactic acid generated over time may have contributed to the reduction of pathogenic microorganism loads measured in the substrate. In particular, the pH parameter can be used as a measure of fermentation efficiency (Deshmukh and Patterson, 1997b). During our study, the production of lactic acid during fermentation of HR mixtures led to a significant reduction in the pH of the residues below values of 5 after 7 days. Being below 5.3, these pH values may be sufficiently acidic to inhibit the growth of certain pathogenic microorganisms such as *Staphylococcus aureus* as it is the case for fermented meat (American Meat Institute, 1982; CFIA, 2020). The acidification process of the HR could be slowed down by the presence of eggshells, which are composed of calcium carbonate with a strong buffering capacity (differential amount of acid/base to be added (dAB)/differential change in pH (dpH) ≃ 1000 equivalent *m*^−3^; Bache, 1984). These conditions mean that higher quantities of organic acids must be produced by lactic acid bacteria to acidify the matrix. In addition to their acidifying power, lactic acid in its undissociated form is soluble to the cytoplasmic membrane of microorganism cells and can interfere with the cell's transmembrane pH gradient, limiting cell development (Wee et al., 2006). During HR fermentation, acetic acid was also produced. This acid can inhibit the development of Gram-positive bacteria (e.g. *Bacillus, Staphylococcus* spp., *Clostridium* spp., *Streptococcus* spp., *Listeria* spp.) and molds, but its effect is more marked on Gram negative bacteria such as enterobacteria and *Pseudomonas* (Reis et al., 2012). The combined production of lactic and acetic acid could therefore explain the reduction in COL loads measured in HR with optimized fermentation.

In our study, the addition of ferment did not appear to be essential for the initiation of fermentation in HR. In fact, in the presence of a fermentable carbohydrate source, LAB loads constituting HR microbiota were sufficient to initiate fermentation, despite variability in microbiological loads of the HR between trials. In food processing, fermentation can occur spontaneously (Lv et al., 2019) or through the addition of ferments, depending on the initial microbiota. Adding ferments, however, can enhance fermentation effects and reduce the risk of process failure (Wang et al., 2013). This was observed in our study, where ferment addition accelerated COL decontamination after 7 days without affecting HR pH. This effect was not due to higher LAB concentrations, as HR were already saturated with LAB (8–9 log cfu/g). Instead, the bioprotective strains constituting the starter culture ferment may have outcompeted the native LAB, modulating the microbiota to accelerate COL reduction. The effectiveness of the inoculated ferment can also be assessed from the results derived from the T treatment. Although the heat process applied did not eliminate the microbiota, it did initially result in a significant reduction in COL loads in HR. This may have reduced competition for the development of LAB inoculated at 7 log cfu/g, whose concentration increased and stabilized at 3 days reaching a stationary phase at 8 to 9 log cfu/g, which is comparable to the LAB loads obtained by Cai et al. (1994) during fermentation of broiler offal. Nevertheless, COL loads measured at 7 and 14 days in T substrates tended to be higher than those in F and S ones. This could be explained by a change in the physical properties due to heat treatment of HR, combined with a reduction in the initial total microbiota load limiting the competitive exclusion capacity of LAB in the substrate. An analysis of the HR 16S ARN genome (Alciatore et al., 2024) could have provided a better understanding of how the HR microbiota was modulated by heating and by the addition of ferment.

In our study, the addition of carbohydrate compounds at optimal inclusion rates (15% for 7-day fermentation, 25-35 % for 14 days) proved crucial for reducing COL contamination but also had an impact on the physico-chemical properties of HR, such as pH, color intensity and odors by limiting the formation of VOCs associated with unpleasant odors in HR. Similarly, Deshmukh and Patterson (1997a) observed a reduction in pH below 5.5 and negligible *E. coli* and *Salmonella* counts when fermenting HR supplemented with chocolate residues (15 %, wet basis) and a commercial ferment, over 21 days. In the same study, a differentiation in color between mixture with low (5%) and high (10-15%) carbohydrate inclusion (greenish brown to dark-brown, pink respectively) and in odor, described as an unpleasant to pleasant, fermented smell, was also noted. The odor improvement was then explained by reduce production of H_2_S and NH_3_ concentrations. These results are in accordance with those obtained by fermenting HR supplemented with whey permeate in our study. Variations in color characteristics measured in both studies could have been caused by the acidification of fermented HR (Swatland, 2008). However, the three times longer fermentation duration to reach these pH and microbiological values in the processes applied by Deshmukh and Patterson (1997a) could be explained by a low initial LAB concentration in HR artificially prepared from fixed ratios of chicks and non-degraded eggs, and a lower fermentability of the selected carbohydrate co-products compared to lactose.

Although an assessment of perceived odor intensity was not performed during our study, the measurement of high concentrations of VOCs, such as dimethyl sulfide (putrid, decayed cabbage odors), dimethyl disulfide (pungent garlic, decaying fish, decomposing materials odors), dimethyl trisulfide (sulfurous, putrid, cabbage, cooked onion odors), trimethylamine (fish and ammonia odors) and indole (feces odors), help define the characteristic unpleasant odors associated with HR (Sazakli and Leotsinidis, 2021). Organic acids produced during optimized HR fermentation may have contributed to their acidic odor, similar to findings by Odey et al. (2018), who reduced odors in human fecal sludge through lactic acid fermentation with carbohydrate supplements. While Zerihun et al. (2021) eliminated fecal odors by fermenting with local carbohydrate co-products, HR fermentation over 14 days did not significantly reduce VOC concentrations but limited their formation in treatments with 15–25% IL compared to those without added lactose. The production of the measured VOC, associated with meat spoilage odors, likely results from amino acid degradation by anaerobic microorganisms when carbohydrate resources are insufficient (Bleicher et al., 2022). The mitigation of these odors through the developed fermentation process will help increase the feasibility and social acceptability of HR bioconversion sites using BSFL.

During HR fermentation, significant variations in pH and coliform loads between treatments were only measured after 7 days, indicating the impact of fermentation time on process efficiency. In the case of fresh HR substrates with 15 % IL, pH is initially reduced below 5 after 7 days but tended to increase again between 7 and 14 days, in contrast to diets with 25-35 % IL. Similarly, the pH of substrates with 0-5% IL increased over two weeks. The increases in pH measured could be explained by the total use of carbohydrate resources, as shown by the absence of glucose detection in substrates with 0-5 % and 15 % IL after 3 and 7 days, respectively. Indeed, in the absence of glucose as primary energy resource, the bacteria will start to degrade nitrogenous compounds, releasing basifying compounds (Kada et al., 2008). As a result, prolonged fermentation without sufficient initial carbohydrates may adversely affect HR stability and reduce their potential for use as a rearing substrate for BSFL. Fermentation over 14 days did not result in a significant reduction in COL loads compared to 7-day fermentation for raw HR at optimized treatments (25-35 % IL). However, a two-week fermentation period could still be a relevant approach to ensure the microbiological quality of HR in a context where their initial contamination would be abnormally high. In this study, D and W whey permeate were also compared as carbohydrate co-products for economic reasons. Since no significant difference was measured between the use of D or W, increasing the water content of the raw HR substrate did not appear to affect process performance. Therefore, both types could represent suitable option for efficient fermentation of HR and the final choice of whey permeate form will depend on its market value and the associated transport costs to the BSFL treatment units.

Fermented HR could be reintroduced into the food production chain by being used as a diet for rearing BSFL for use in animal feed (Dallaire-Lamontagne et al., 2024). Although HR is a protein- and lipid-rich co-product and its optimized fermentation effectively reduces COL loads, it may not meet regulatory standards for insect rearing substrates. In Canada, insects can be used as feed but are not considered livestock, and the registration requirements for feed ingredients derived from insects do not specify permissible substrates (CFIA, 2019). Nevertheless, hazards in the substrate must be identified, and the final insect feed must be free from harmful microorganisms for registration and commercialization (CFIA, 2021). Thus, it should be considered that the absence of COL detection in optimally fermented HR does not exclude the presence of other potentially pathogenic microorganisms that were not measured in this study, such as *L. monocytogenes*. In addition, even if the substrate has been treated upstream, larvae may be exposed to recontamination as they evolve directly in contact with their diet and feces (Vandeweyer et al., 2021). In the case of bacterial spores, they are not inactivated by fermentation, but the acidic conditions of fermented substrates can restrict their development (Klunder et al., 2012). However, BSFL tend to raise the pH of their diet during the bioconversion process (Meneguz et al., 2018), which could make the conditions for spore germination favourable again. These considerations could imply that subsequent quality control treatments will have to be applied to the final insect products even with initial fermentation of the diets (Saucier et al., 2021). Before considering the commercialization of larvae and frass derived from fermented HR, the microbiological risks will need to be assessed on the final products. The impact of modifying the pH and microbiota of fermented HR on the rearing of BSFL will also need to be evaluated, as these parameters can affect their bioconversion performances (Auger et al., 2023; Ma et al., 2018).

In conclusion, this study shows that fermentation can effectively reduce some of the risks associated with HR, upstream of their use as a rearing diet for BSFL. However, the fermentation process must be carried out over a minimum period of 7 days and be optimized by adding whey permeate as a carbohydrate source, at lactose inclusion level of 15 to 35 %. It can also benefit from the inoculation of a ferment with bioprotective effects. The result led to a significant acidification of HR, a reduction in COL and *E. coli* loads below detection thresholds, and limitation of the formation of VOCs associated with unpleasant odors. These conditions will enable better stabilization of HR over time, as well as improved acceptability of their use as BSFL feed substrate. The process could be adapted by modulating fermentation time, carbohydrate content and ferment addition according to variations in HR composition and associated microbiological and odor mitigation. However, before applying the process, several parameters will require further assessment to ensure its feasibility. These include evaluating larval capacity for bioconversion of fermented HR, verifying that resulting larvae and frass meet regulatory quality standards, and evaluate economic viability of the system.

## Role of the funding source

This project received support from Couvoir Scott Ltée and the Alliance program of the National Sciences and Engineering Research Council of Canada (Award Number: ALLRP – 568489-21). Couvoir Scott Ltée provided hatchery residues, infrastructures, and personnel for the preparation of the experiments. The funding sources had no others involvement in the conduct of the research and the preparation of the article.

## Declaration of competing interest

The authors declare the following financial interests or personal relationships which may be considered potential competing interests: Marie-Hélène Deschamps reports financial support provided by Couvoir Scott Ltée. Mariève Dallaire-Lamontagne reports a consulting or advisory relationship with Couvoir Scott Ltée. Jean-Michel Allard Prus and Jérémy Lavoie reports employment with Couvoir Scott Ltée. Any other authors declare that they have no known competing financial interests or personal relationships that could have influenced the work reported in this paper.

## Appendix Supplementary materials

Image, application 1

## References

[bib0001] Alciatore G., Peguero D.A., Gold M., Zurbrügg C., Niu M., Bargetze F., Mathys A. (2024). Preservation of agri-food byproducts by acidification and fermentation in black soldier fly larvae bioconversion. Waste Manage.

[bib0002] American Meat Institute (1982).

[bib0004] Auger L., Bouslama S., Deschamps M.H., Vandenberg G., Derome N. (2023). Absence of microbiome triggers extensive changes in the transcriptional profile of *hermetia illucens* during larval ontology. Sci. Rep..

[bib0005] Baba I.A., Banday M.T., Khan H.M., Khan A.A., Sheikh I.U.D. (2022). Effect of Lactobacillus acidophilus (LB) and saccharomyces cerevisiae (Yeast) fermentation on nitrogen, phosphorus and potassium level of poultry farm waste. Ecol. Environ. Conserv..

[bib0006] Bache B.W. (1984). The role of calcium in buffering soils. Plant Cell Environ..

[bib0007] Bartkiene E., Bartkevics V., Mozuriene E., Lele V., Zadeike D., Juodeikiene G. (2019). The safety, technological, nutritional, and sensory challenges associated with lacto-fermentation of meat and meat products by using pure lactic acid bacteria strains and plant-lactic acid bacteria bioproducts. Front. Microbiol..

[bib0008] Benchaar C., Hassanat F., Gervais R., Chouinard P.Y., Julien C., Petit H.V., Massé D.I. (2013). Effects of increasing amounts of corn dried distillers grains with solubles in dairy cow diets on methane production, ruminal fermentation, digestion, N balance, and milk production. J. Dairy Sci..

[bib0009] Bleicher J., Ebner E.E., Bak K.H. (2022). Formation and analysis of volatile and odor compounds in meat—A review. Molecules.

[bib0010] Borremans A., Bußler S., Sagu S.T., Rawel H., Schlüter O.K., Leen V.C. (2020). Effect of blanching plus fermentation on selected functional properties of mealworm (*Tenebrio molitor*) powders. Foods.

[bib0011] Bosch G., Swanson K.S. (2020). Effect of using insects as feed on animals: pet dogs and cats. J. Insects Food Feed.

[bib0012] Bruins M.E., Sanders J.P.M. (2012). Small-scale processing of biomass for biorefinery. Biofuels Bioprod. Biorefin..

[bib0013] Cai T., Pancorbo O.C., Barnhart H.M., Sander J.E., Merka W.C. (1994). Chemical and microbiological characteristics of poultry processing by-products, waste, and poultry carcasses during lactic acid fermentation. J. Appl. Poult. Res..

[bib0014] Casaburi A., Piombino P., Nychas G.J., Villani F., Ercolini D. (2015). Bacterial populations and the volatilome associated to meat spoilage. Food Microbiol..

[bib0015] Castellano P., Belfiore C., Fadda S., Vignolo G. (2008). A review of bacteriocinogenic lactic acid bacteria used as bioprotective cultures in fresh meat produced in Argentina. Meat Sci..

[bib0016] Castilho N.P.A., Colombo M., De Oliveira L.L., Todorov S.D., Nero L.A. (2019). Lactobacillus curvatus UFV-NPAC1 and other lactic acid bacteria isolated from calabresa, a fermented meat product, present high bacteriocinogenic activity against *Listeria monocytogenes*. BMC Microbiol..

[bib0017] CFIA. 2019. Registration requirements for insect-derived livestock feed ingredients. Accessed April 2024. https://inspection.canada.ca/animal-health/livestock-feeds/consultations/registration-requirements/eng/1557837434904/1557837435158.

[bib0018] CFIA. 2020. Preventive control recommendations for manufacturing fermented and dried meat products. Accessed July 2024. https://inspection.canada.ca/en/preventive-controls/meat/fermented-and-dried.

[bib0019] CFIA. 2021. Proposed document to be incorporated by reference – Canadian Feed Ingredients Table (CFIT). Accessed April 2024. https://inspection.canada.ca/animal-health/livestock-feeds/regulatory-modernization/cfit/eng/1613423298011/1613423298323.

[bib0021] Dallaire-Lamontagne M., Lebeuf Y., Allard Prus J.M., Vandenberg G.W., Saucier L., Deschamps M.H. (2024). Characterization of hatchery residues for on farm implementation of circular waste management practices. Waste Manage.

[bib0022] Deshmukh A.C., Patterson P.H. (1997). Preservation of hatchery waste by lactic acid fermentation. 1. Laboratory scale fermentation. Poult. Sci..

[bib0023] Deshmukh A.C., Patterson P.H. (1997). Preservation of hatchery waste by lactic acid fermentation. 2. Large-scale fermentation and feeding trial to evaluate feeding value. Poult. Sci..

[bib0024] Dicks L.M.T., Mellett F.D., Hoffman L.C. (2003). Use of bacteriocin-producing starter cultures of *Lactobacillus plantarum* and *Lactobacillus curvatus* in production of ostrich meat salami. Meat Sci..

[bib0025] Eissa A.E., Yusuf M.S., Younis N.A., Fekry M., Dessouki A.A., Ismail G.A., Ford H., Abdelatty A.M. (2022). Effect of poultry offal silage with or without betaine supplementation on growth performance, intestinal morphometry, spleen histomorphology of Nile tilapia (*Oreochromis niloticus*) fingerlings. J. Anim. Physiol. Anim. Nutr..

[bib0026] García-Díez J., Saraiva C. (2021). Use of starter cultures in foods from animal origin to improve their safety. Int. J. Environ. Res. Public Health.

[bib0027] Gill C.O. (2000).

[bib0028] Di Gioia D., Mazzola G., Nikodinoska I., Aloisio I., Langerholc T., Rossi M., Raimondi S., Melero B., Rovira J. (2016). Lactic acid bacteria as protective cultures in fermented pork meat to prevent *Clostridium* spp. Growth. Int. J. Food Microbiol..

[bib0029] Gooding C.H. (2012). Data for the carbon footprinting of rendering operations. J. Ind. Ecol..

[bib0030] Gouvernement du Québec. 2022. Politique québécoise de gestion des matières organiques résiduelles; Q-2, R. 35.1. Accessed December 2022. https://www.legisquebec.gouv.qc.ca/fr/document/rc/Q-2,%20r.%2035.1.

[bib0031] Health Canada. 2001. Determination of the aerobic colony count of foods, MFHPB-18. Accessed June 2022. https://www.canada.ca/en/health-canada/services/food-nutrition/research-programs-analytical-methods/analytical-methods/compendium-methods/methods-microbiological-analysis-foods-compendium-analytical-methods.html.

[bib0032] Health Canada. 2016. Enumeration of Escherichia coli and Coliforms in Food Products and Food Ingredients Using 3M™ Petrifilm™ E. coli Count Plates, MFHPB-34. Ottawa, ON, Canada. Accessed June 2022. https://www.canada.ca/en/health-canada/services/food-nutrition/research-programs-analytical-methods/analytical-methods/compendium-methods/methods-microbiological-analysis-foods-compendium-analytical-methods.html.

[bib0033] Hutchison M.L., Walters L.D., Avery S.M., Munro F., Moore A. (2005). Analyses of livestock production, waste storage, and pathogen levels and prevalences in farm manures. Appl. Environ. Microbiol..

[bib0034] IPIFF. 2024. EU legislation – General overview. Accessed April 2024. https://ipiff.org/insects-eu-legislation-general/.

[bib0035] Kada S., Yabusaki M., Kaga T., Ashida H., Yoshida K.I. (2008). Identification of two major ammonia-releasing reactions involved in secondary natto fermentation. Biosci. Biotechnol. Biochem..

[bib0036] Klunder H.C., Wolkers-Rooijackers J., Korpela J.M., Nout M.J.R. (2012). Microbiological aspects of processing and storage of edible insects. Food Control.

[bib0037] Lähteenmäki-Uutela A., Hénault-Ethier L., Marimuthu S.B., Talibov S., Allen R.N., Nemane V., Vandenberg G.W., Józefiak D. (2018). The impact of the insect regulatory system on the insect marketing system. J. Insects Food Feed.

[bib0038] Larouche J., Campbell B., Hénault-Éthier L., Banks I.J., Tomberlin J.K., Preyer C., Deschamps M.H., Vandenberg G.W. (2023). The edible insect sector in Canada and the United States. Anim. Front..

[bib0039] Lv J., Yang Z., Xu W., Li S., Liang H., Ji C., Yu C., Zhu B., Lin X. (2019). Relationships between bacterial community and metabolites of sour meat at different temperature during the fermentation. Int. J. Food Microbiol..

[bib0040] Ma J., Lei Y., ur Rehman K., Yu Z., Zhang J., Li W., Li Q., Tomberlin J.K., Zheng L. (2018). Dynamic effects of initial pH of substrate on biological growth and metamorphosis of black soldier fly (Diptera: stratiomyidae). Environ. Entomol..

[bib0041] Meneguz M., Gasco L., Tomberlin J.K. (2018). Impact of pH and feeding system on BSF larval development. PLoS One.

[bib0042] Nahariah N., Legowo A.M., Abustam E., Hintono A. (2015). Angiotensin I-converting enzyme inhibitor activity on egg albumen fermentation. Asian-Australas. J. Anim. Sci..

[bib0043] Odey E.A., Li Z., Zhou X., Yan Y. (2018). Locally produced lactic acid bacteria for pathogen inactivation and odor control in fecal sludge. J. Clean. Prod..

[bib0044] Orrico A.C.A., Schwingel A.W., de M.S.S., Costa M., Orrico Junior M.A.P., Borquis R.R.A., Alves G.P., de Oliveira J.D., Leite B.K.V., Garcia R.G., da R.N., Vilela S. (2020). Characterization and valuing of hatchery waste from the broiler chicken productive chain. Waste Manage.

[bib0046] Prudêncio C.V., dos Santos M.T., Vanetti M.C.D. (2015). Strategies for the use of bacteriocins in gram-negative bacteria: relevance in food microbiology. J. Food Sci. Technol..

[bib0047] Raa J., Gildberg A. (1982). Fish silage: a review. Crit. Rev. Food Sci. Nutr..

[bib0048] Reis J.A., Paula A.T., Casarotti S.N., Penna A.L.B. (2012). Lactic acid bacteria antimicrobial compounds: characteristics and applications. Food Eng. Rev..

[bib0050] Samer M., Abdelsalam E.M. (2019).

[bib0051] Saucier L., Gendron C., Gariépy C. (2000). Shelf life of ground poultry meat stored under modified atmosphere. Poult. Sci..

[bib0052] Saucier L., M'ballou C., Ratti C., Deschamps M.H., Lebeuf Y., Vandenberg G.W. (2021). Comparison of black soldier fly larvae pre-treatments and drying techniques on the microbial load and physico-chemical characteristics. J. Insects Food Feed.

[bib0053] Sazakli E., Leotsinidis M. (2021). Odor nuisance and health risk assessment of VOC emissions from a rendering plant. Air. Qual. Atmos. Health.

[bib0056] Solomons N.W. (2002). Fermentation, fermented foods and lactose intolerance. Eur. J. Clin. Nutr..

[bib0057] Swatland H.J. (2008). How pH causes paleness or darkness in chicken breast meat. Meat Sci..

[bib0058] Vandeweyer D., De Smet J., Van Looveren N., Van Campenhout L. (2021). Biological contaminants in insects as food and feed. J. Insects Food Feed.

[bib0059] Veldkamp T., Vernooij A.G. (2021). Use of insect products in pig diets. J. Insects Food Feed.

[bib0062] Wang X.H., Ren H.Y., Liu D.Y., Zhu W.Y., Wang W. (2013). Effects of inoculating *Lactobacillus sakei* starter cultures on the microbiological quality and nitrite depletion of Chinese fermented sausages. Food Control.

[bib0063] Wee Y.J., Kim J.N., Ryu H.W. (2006). Biotechnological production of lactic acid and its recent applications. Food Technol. Biotechnol..

[bib0064] Zerihun G., Agizew N., Adey D., Nancy G.L. (2021). Food waste produced lactic acid for pathogen inactivation, urea stabilization and odor control in feces. Afr. J. Environ. Sci. Technol..

[bib0065] Zhang J., Zhang J., Li J., Tomerlin J.K., Xiao X., ur Rehman K., Cai M., Zheng L., Yu Z. (2021). Black soldier fly: a new vista for livestock and poultry manure management. J. Integr. Agric..

