# Supplementary material for: Optimization of a hatchery residue fermentation process for potential recovery by black soldier fly larvae
Source: Poult Sci. 2025 Feb 25;104(4):104946. doi: 10.1016/j.psj.2025.104946 (PMC11926703; doi:10.1016/j.psj.2025.104946)
Supplement: Supplementary file 1 [file mmc1.docx]

**SUPPLEMENTARY MATERIAL**

Table S1. Pearson correlation coefficient (r) between day of fermentation (d), lactose inclusion level (IL) and parameters measured during fermentation of hatchery residue.

|  | d | IL | pH | Chroma | TAM | LAB | COL | *E. coli* | Lactic | Acetic |
| --- | --- | --- | --- | --- | --- | --- | --- | --- | --- | --- |
| d | 1.00 | 0.00 | -0.43 | 0.47 | -0.24 | -0.08 | **-0.52** | **-0.53** | **0.58** | 0.46 |
| IL | 0.00 | 1.00 | **-0.50** | 0.13 | 0.02 | 0.07 | -0.19 | -0.17 | 0.47 | -0.10 |
| pH | -0.43 | **-0.50** | 1.00 | -0.41 | 0.02 | -0.16 | 0.27 | 0.17 | **-0.65** | -0.23 |
| Chroma | 0.47 | 0.13 | -0.41 | 1.00 | 0.22 | 0.28 | -0.16 | -0.20 | 0.36 | 0.06 |
| TAM | -0.24 | 0.02 | 0.02 | 0.22 | 1.00 | **0.71** | 0.49 | 0.43 | -0.11 | -0.21 |
| LAB | -0.08 | 0.07 | -0.16 | 0.28 | **0.71** | 1.00 | 0.48 | 0.44 | 0.02 | -0.11 |
| COL | **-0.52** | -0.19 | 0.27 | -0.16 | 0.49 | 0.48 | 1.00 | **0.96** | **-0.52** | -0.19 |
| *E. coli* | **-0.53** | -0.17 | 0.17 | -0.20 | 0.43 | 0.44 | **0.96** | 1.00 | -0.48 | -0.15 |
| Lactic | **0.58** | 0.47 | **-0.65** | 0.36 | -0.11 | 0.02 | **-0.52** | -0.48 | 1.00 | 0.27 |
| Acetic | 0.46 | -0.10 | -0.23 | 0.06 | -0.21 | -0.11 | -0.19 | -0.15 | 0.27 | 1.00 |

Red colour indicated a strong positive correlation (r > 0.5). Blue colour indicated a strong negative correlation (r < -0.5). TAM = total aerobic mesophilic; COL = coliforms; LAB = presumptive lactic acid bacteria. Lactic = lactic acid; Acetic = acetic acid.

Table S2. Table of Abbreviations

| Abbreviation | Definition |
| --- | --- |
| HR | Hatchery residues |
| BSFL | Black soldier fly larvae |
| Su | Hatchery residues substrate |
| S | Treatment (spontaneous fermentation): raw HR without addition of a ferment |
| F | Treatment (inoculated fermentation): raw HR with ferment (0.3%, wet basis) |
| T | Treatment (thermal pre-treatment): thermally treated HR with ferment (0.3%, wet basis) |
| IL | Lactose inclusion level to HR (%, dry basis) |
| D | Dry form of whey permeate |
| W | Wet form of whey permeate |
| VFAs | Volatile fatty acids |
| VOCs | Volatile organic compounds |
| TAM | Total aerobic mesophilic bacteria |
| LAB | Lactic acid bacteria |
| COL | Coliforms |

Table S3. List of materials, reagents, and equipment used in the experimental procedures

| Section | Material/Equipment | Commercial Name | Supplier | Location |
| --- | --- | --- | --- | --- |
| Hatchery Residues | Sealed plastic containers | S-15637BLU | Uline | Pleasant Prairie, WI |
|  | Meat grinder | #12 Big Bite Meat Grinder | LEM | OH, USA |
|  | Drying oven | VWR® Forced Air Ovens | VWR International | Radnor, PA, USA |
| Experimental Design | Freeze-dried commercial ferment | LALCULT® Protect SAX-01 | Lallemand | Montréal, Canada |
|  | Commercial fermentation jars | Premium E-jen Kimchi Fermentation & Storage Container | Crazy Korean Cooking | Ridgefield Park, NJ |
|  | Whey permeate (dry or wet form) | - | Agropur | Saint-Hyacinthe, Qc, Canada |
|  | Vacuum-sealed bags | Smooth Vacuum Bags SB Series | Sous Vide Premium | Montréal, Canada |
|  | Vacuum sealer | Eco Vacuum | Orved Spa | Venice, Italy |
|  | Pressure cooker | Induction Compatible Pressure Canner 01784 | National Presto Industries | Eau Claire, WI, USA |
| Sampling and monitoring | Portable pH meter | Orion Star™ A221 | Thermo Fisher Scientific | Waltham, MA, USA |
|  | pH probe | Orion™ Triode™ 9107BNMD | Thermo Fisher Scientific | Waltham, MA, USA |
|  | Colorimeter | Chromameter CR400/410 | Konica Minolta | Tokyo, Japan |
|  | Colorimetric strips | MQuant™ Glucose Test Strips | MilliporeSigma™ | Burlington, MA, USA |
| Microbiological analyses | Buffered peptone water | Difco™ Buffered Peptone Water | Difco Laboratories Inc., Becton Dickinson | Franklin Lakes, NJ, USA |
|  | Stomacher | Stomacher® 400C | Seward Laboratory Systems Inc. | London, UK |
|  | Plate Count Agar medium | BD Difco™ Plate Count Agar | Difco Laboratories Inc. | Franklin Lakes, NJ, USA |
|  | De Man, Rogosa, and Sharp agar | BD Difco™ Lactobacilli MRS Broth | Difco Laboratories Inc. | Franklin Lakes, NJ, USA |
|  | Anaerobic container system | GasPak EZ container system | Becton Dickinson | Franklin Lakes, NJ, USA |
|  | Petrifilms (E. coli/Coliform) | 3M™ Petrifilm™ Rapid E. coli/Coliform Count Plate | 3M | Montréal, Canada |
| Volatile fatty acids (VFAs) | Resin (AG 50WX8, 100–200 Mesh, H) | BioRad | BioRad | Hercules, CA, USA |
|  | HPLC systems | 1260 Infinity II LC System | Agilent | Santa Clara, CA, USA |
| Volatile organic compounds (VOCs) | SPME fibre | Supelco 57299-U, 50/30 µm DVB/CAR/PDMS, 2 cm StableFlex | Supelco Inc. | Bellefonte, PA, USA |
|  | Autosampler for SPME | Combi PAL Autosampler | CTC Analytics | Zwingen, Switzerland |
|  | Gas Chromatograph (GC) | Agilent 8890 GC | Agilent Technologies Canada Inc. | Mississauga, Canada |
|  | Mass Spectrometer (MS) | Agilent 5977B GC/MSD | Agilent Technologies Canada Inc. | Mississauga, Canada |
|  | Internal standard (hexanal-δ12) | - | C/D/N Isotopes Inc. | Pointe-Claire, Canada |
|  | Optima Wax column | 60 m x 250 µm x 0.25 µm | Macherey-Nagel | Düren, Germany |
